# Staged transverse preputial island flap urethroplasty for some proximal hypospadias with moderate-to-severe chordee

**DOI:** 10.1186/s12894-021-00948-8

**Published:** 2021-12-23

**Authors:** Hai Lin, Yu-Yun Wang, Shi-Bing Li, Ze-Ting Chen, Liang-Ju Su

**Affiliations:** Department of Pediatric Surgery, Hainan General Hospital (Hainan Affiliated Hospital of Hainan Medical University), Xiuying District, Haikou, 570311 Hainan People’s Republic of China

**Keywords:** Proximal hypospadias, Staged, Urethroplasty, Surgical outcome

## Abstract

**Background:**

We aimed to assess the outcome of staged transverse preputial island flap (TPIF) urethroplasty for repairing certain cases of primary proximal hypospadias with moderate-to-severe chordee in children.

**Methods:**

Nighty-two consecutive boys who underwent either one-stage or staged TPIF urethroplasty for the repair of proximal hypospadias with moderate-to-severe chordee between August 2015 and December 2019 were evaluated retrospectively. Patients were divided into two groups: one-stage TPIF urethroplasty group (n = 44) and staged TPIF urethroplasty group (n = 48). We noted and compared the postoperative complications, including urethrocutaneous fistula, urethral diverticula, residual penile curvature, and urethral stricture in both groups.

**Results:**

Both groups were followed up for 1–5 years, with an average of 3 years. No cases of residual or recurrence of penile chordee were reported in either group. In Group A, 9 patients (9/44, 20.4%) had postoperative urethrocutaneous fistula, and all patients underwent urinary fistula repair or urethroplasty. In Group B, postoperative urethrocutaneous fistula occurred in 2 cases (2/48, 4.1%), and one patient developed a urethrocutaneous fistula after the first operation, which was successfully repaired during the second operation. A urethrocutaneous fistula occurred in 1 case after completion of the second-stage operation; urethral fistula repair was performed successfully 6 months later. There were 2 cases of urethral stricture in Group A (2/44, 4.5%) and none in Group B. There were 6 cases of urethral diverticulum in Group A (6/44, 13.6%) and no cases of urethral diverticulum in Group B. The operative success rates were 61.3% and 95.8% in Group A and Group B, respectively (*P* < 0.001).

**Conclusions:**

Compared with one-stage TPIF urethroplasty, staged TPIF urethroplasty in the treatment of certain cases of primary proximal hypospadias with moderate-to-severe chordee resulted in fewer postoperative fistulas, urethral strictures and urethral diverticula. The staged TPIF urethroplasty procedure was effective in reducing the operation difficulty and complication rate of hypospadias, improving the curative effect of complex hypospadias and having good clinical application value.

## Background

Which operative technique can we choose to repair severe hypospadias? It depends on the severity of hypospadias and the experience of the surgeon. Severe hypospadias is often accompanied by severe malformations and limited repair materials, which will lead to more postoperative complications. Proximal hypospadias [1] is defined by an external orifice of the urethra located on the proximal penile shaft to perineum at the beginning of urethroplasty after the chordee is corrected. “Severe” hypospadias specifically refers to cases with ≥ 30° chordee after degloving. Staged surgery is often required, and satisfactory efficacy and good cosmesis still cannot be achieved. Therefore, the treatment of severe hypospadias is a great challenge for pediatric urologists. There are different opinions on the choice of single-stage versus staged operations [2, 3]. We believe that the experience of the surgeon and the severity of hypospadias are the main determinants. We aimed to assess the outcome of staged transverse preputial island flap (TPIF) urethroplasty for repairing certain cases of primary proximal hypospadias with moderate-to-severe chordee in children. We have performed one-stage and two-stage urethroplasty combining transected urethral plate and TPIF to treat initially severe hypospadias in our hospital since 2015, with two-stage procedures producing desirable effects.

## Methods

The study was conducted in accordance with the Declaration of Helsinki (as revised in 2013). The study was approved by the ethics board of Hainan General Hospital (2021-005) and informed consent was obtained from a parent or guardian for participants under 16 years old.

### Patient criteria

**Fig. 1:**
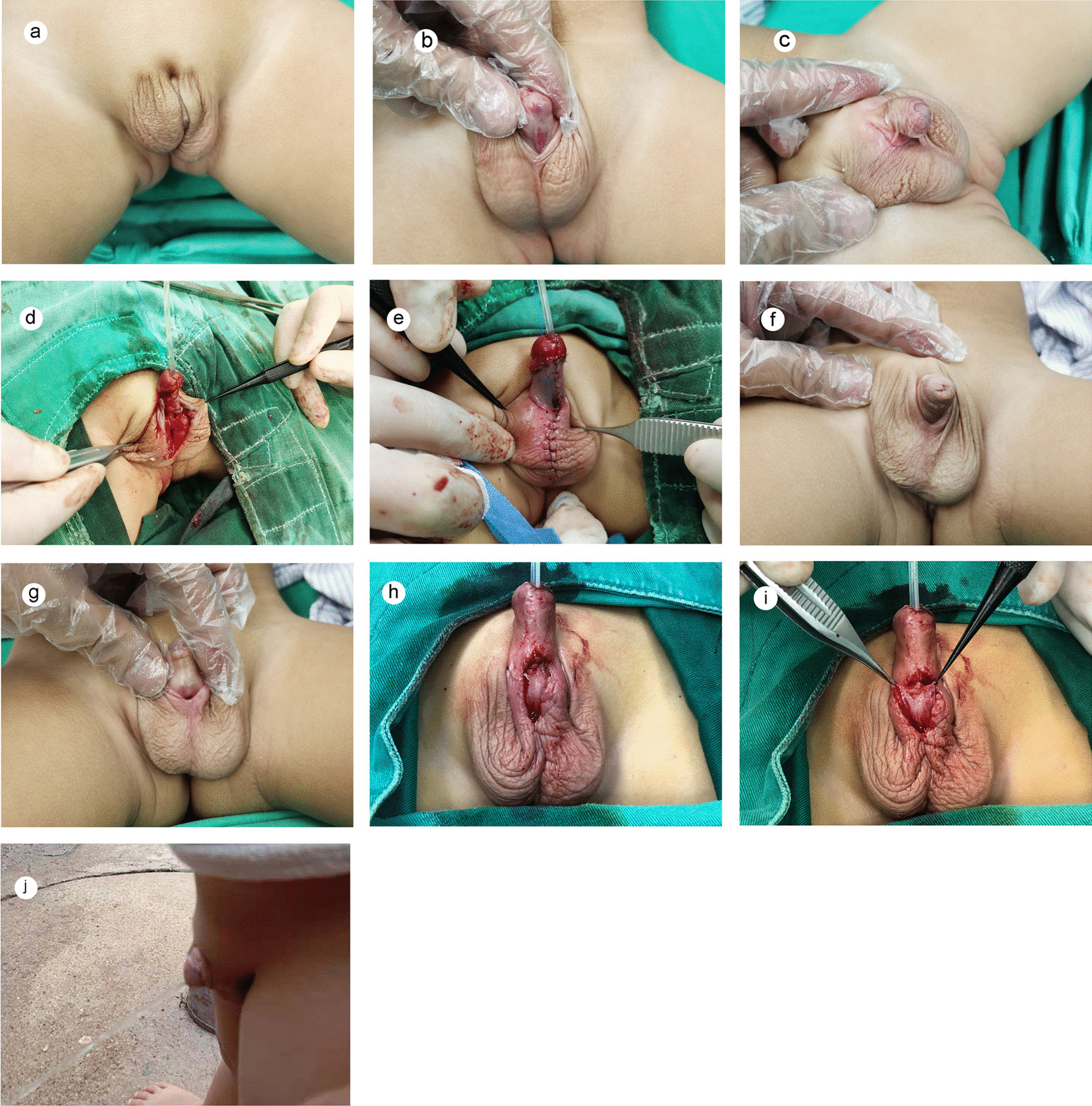
**a**–**c** Front and lateral views before stage I repair. Bilateral scrotal separation, severe chordee of the penis, urethra external orifices located at the penile scrotal junction. **d** The island flap was sutured to form a tube and transposed ventrally. The distal end of the neourethra was sutured with the penile head, and the two wings of the penile head were closed. The proximal end of the formed urethral tube was anastomosed with the urethral plate. **e** Appearance immediately after stage I repair. Byar’s flaps were created and swung ventrally to cover the ventral defect, and the ventral side of the anastomosis was sutured with the skin around the scrotum to form the stoma. **f**, **g** Appearance before stage II repair. The penis was straightened satisfactorily, and the fistula was located at the junction of the penis and scrotum. **h** The urethral stoma formed in the first stage was dissociated, and the urethra was sutured. **i** Ventrally transposed thick and healthy dartos tissue near the fistula was used to cover the neourethra. **j** State during urination 3 months after stage II repair. The patient achieved an apical slit-like meatus at the tip of the glans penis, a single forward stream, unimpeded voiding, and good cosmesis

Ninety-two children underwent their first surgery at the average age of 18 months. All were penoscrotal hypospadias or perineal hypospadias. Forty-four patients underwent one-stage TPIF urethroplasty (Group A), including 10 patients with penoscrotal hypospadias and 34 patients with perineal hypospadias. Forty-eight patients underwent staged TPIF urethroplasty (Group B), including 20 patients with penoscrotal hypospadias (Fig. 1a–c) and 28 patients with perineal hypospadias, and the interval between the two stages was at least 6 months. The preoperative data of the patients are summarized in Table 1.

**Table 1:** Basic characteristics of patient

	One-stage (n = 44)	Two-stage (n = 48)
*Location of the meatus (n)*		
Penoscrotal	10	20
Perineal	34	28
*Chordee (n)*		
Severe angulation (> 45°)	30	38
Moderate angulation (> 10°, < 45°)	14	10
*Surgical ages (months, median [IQR])*		
Age at the first stage	18	18
Age at the second stage	–	24
Time between stages	–	10

Data are expressed as number of patients

### Operative technique

The same surgical team performed all the operations. The procedure was divided into two phases. The first phase was performed with chordee correction and partial urethroplasty with a dorsal transverse pedicled island flap, leaving a fistula at the scrotum. The second phase was performed with urethral fistula repair at least 6 months later.

### Single-stage repair

#### Chordee correction

In the first stage, 5/0 polypropylene was sutured longitudinally through the tip of the glans for traction, and a circumferential incision was made 3 mm below the corona, preserving the urethral plate. Then, we excised the whole ventral bands by degloving the penile skin to the root. Next, an artificial erection test (a tourniquet was bonded at the root of the penis, and a butterfly needle was inserted into the tip of the penis. Normal saline solution was intermittently injected into the corpus cavernosum during the operation to evaluate the degree of penile chordee.) was performed. The urethral plate was transected at the corona if the penis could not be straightened. Then, the artificial erection test was repeated. Dorsal plication using 5–0 polypropylene was performed to gain a straight penis when residual chordee remained. The artificial erection test was performed again.

#### Urethroplasty

Next, the free residual urethral plate with a length of approximately 1.0–1.5 cm was fixed on the cavernosal body without tension. The dorsal prepuce inner plate of the penis was cut horizontally, and the vascular pedicle of the island flap was separated to the prepuce root. The length of the island flap was consistent with the length of the urethra, and the width was approximately 1.0–1.2 cm. The pedicled island skin flap was wrapped around a 6–8 F silicone catheter to form a tubular urethra, and 6/0 monofilaments were sutured continuously and transposed ventrally.

#### Meatoplasty and glanuloplasty

The ventral side of the glans was split longitudinally in the midline from the subcoronal sulcus to the tip, and both sides of the glanular wings were dissociated. The distal end of the neourethra was sutured with the penile head, and the two wings of the penile head were closed (Fig. 1d). The proximal end of the neourethra was sutured with the urethral plate. The dorsal prepuce was transferred to cover the ventral defect.

#### Scrotoplasty

The extra skin of the penile scrotum was cut to form the penile scrotum, and a Y-shaped incision at the junction of the penile scrotum was made to correct the transposition of the penile scrotum.

### Postoperative management

The child was discharged home with a catheter on the fifth day after surgery, and the catheter remained in place for 21 days after the operation. Intravenous antibiotics were administered for 3 days and then changed to oral antibiotics until the catheter was removed.

### Two-stage repair

#### Partial urethroplasty and urethrostomy

The procedure of the first stage was almost the same as that of single-stage urethroplasty. After the proximal end of the formed urethral tube was anastomosed with the urethral plate, the ventral side of the anastomosis was sutured with the skin around the scrotum to form the stoma (Fig. 1e). The stoma was placed as far as possible into the scrotum.

#### Urethral fistula repair/urethroplasty

We performed the second-stage surgery at least 6 months after the initial operation, after edema subsided and healing was adequate. A 5/0 polypropylene was sutured longitudinally through the tip of the glans for traction. Artificial erection was performed as the first step to ensure that there was no residual chordee. The second step was to detect urethrocutaneous fistulae and urethral diverticulum in the distal urethra by water injection and to investigate urethral strictures with a urethral probe. The stoma was closed by annular incision, and the skin of the fistula was sutured by double-layer varus (Fig. 1h).

### Protective intermediate layer

**Fig. 2:**
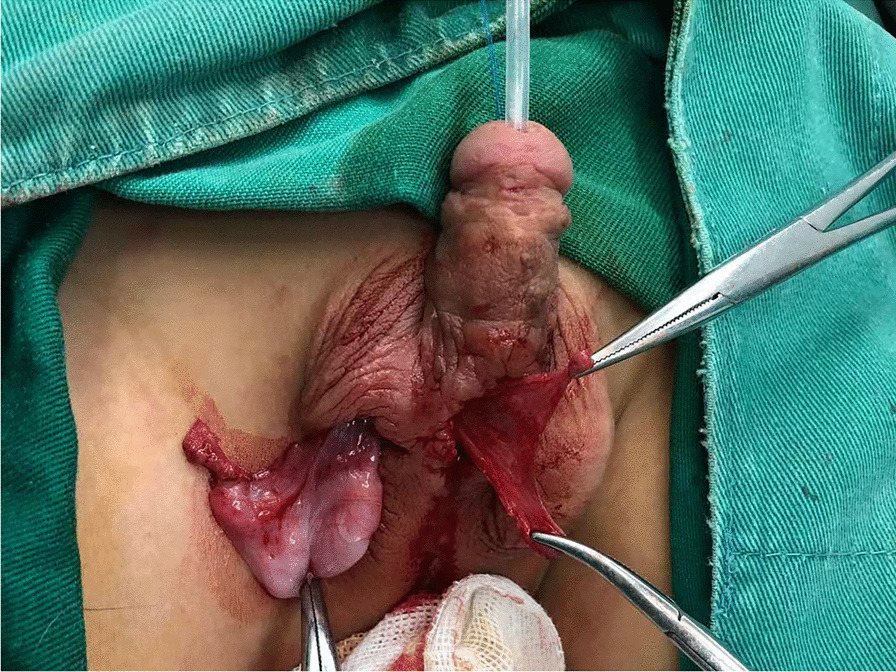
In cases of insufficient or thin dartos tissue, a healthy and thick tunica vaginalis flap was used as a second layer of coverage for the neourethra (another patient)

A ventrally transposed thick and healthy dartos tissue near the fistula was used to cover the neourethra (Fig. 1i). In cases of insufficient or thin dartos tissue, a healthy and thick tunica vaginalis flap was used as a second layer of coverage (Fig. 2).

### Postoperative management

The child was discharged home with a catheter on the fifth day after surgery, and the catheter was withdrawn 21 days postoperatively. Intravenous antibiotics were administered for 3 days and then changed to oral antibiotics until the catheter was removed.

### Statistical analysis

Statistical differences in the detection rates of postoperative incidences of penile chordee, urethrocutaneous fistula, urethral stricture, and urethral diverticulum between the two groups were compared using the chi-squared test. SPSS software (Version 25.0, Chicago, IL) was used for the statistical analysis. Data were considered significant if the *p*-value < 0.05.

### Follow-up

Postoperative follow-up included answering inquiries about the voiding pattern from parents by phone calls or WeChat; physical examination of the genitalia; and direct observation of the voiding stream by the doctor (after removal of the catheter, at 3, 6 and 12 months).

## Results

A total of 92 patients who underwent either one-stage or staged TPIF urethroplasty were identified. All patients were followed up for more than 12 months after the operation. Both groups were followed up for 1 to 5 years, with an average of 3 years. No cases of residual or recurrence of penile chordee were reported in the two groups. All patients achieved good cosmesis and normal urination.

During the follow-up period, the postoperative complication rates were 38.6% and 4.2% in Group A and Group B, respectively. 9 patients (9/44, 20.4%) in Group A developed postoperative urethrocutaneous fistula, which was located at the anastomosis of the old and new urethra. Fistula repair was performed 1 year after the first operation, and no recurrence of fistula occurred. In Group B, postoperative urethrocutaneous fistula occurred in 2 cases (2/48, 4.1%), and one patient developed a urethrocutaneous fistula after the first operation, which was successfully repaired during the second operation. A urethrocutaneous fistula occurred in 1 case after completion of the second-stage operation, and urethral fistula repair was performed successfully 6 months later.There was statistical significance in the incidence of urethrocutaneous fistula between the two groups (*P* = 0.016).

There were 2 cases of urethral stricture in Group A (2/44, 4.5%) and none in Group B. Urethral dilation was not routinely performed between two surgeries in Group B. There was no significant difference between the two groups (*p* = 0.226). In Group A, 2 cases of urethral stricture appeared 1–2 months after the operation. One case achieved good results in urination 1 month after urethral dilation and an indwelling urethral catheter was performed. One patient underwent urethrostomy and two-stage urethroplasty due to failure of urethral dilation, and 1 was cured. No recurrent urethral stricture occurred.

There were 6 cases of urethral diverticulum in Group A (6/44, 13.6%) and no cases of urethral diverticulum in Group B. There was statistical significance in the incidence of urethral diverticulum between the two groups (*P* = 0.026). Six cases of urethral diverticulum in Group A underwent diverticulectomy and urethroplasty 6 months after the operation. Five cases healed. One patient had a urethrocutaneous fistula after surgery and was cured by fistula repair 1 year later. The operative success rates were 61.4% and 95.8% in Group A and Group B, respectively (*P* < 0.001) (Table 2).

**Table 2:** Comparison of postoperative complications and success rate

Treatment	Success	Failure			
		Penis curvature	Fistula	Stricture	Diverticulum
One-stage	27 (44)	0	9	2	6
Two-stage	46 (48)	0	2	0	0
*P* value	< .001	NS	.016	.226	.026

Data are expressed as the number of patients, and *P* values were calculated for the comparison of postoperative complications and success rates between one-stage and two-stage treatment

## Discussion

The debate over repair of proximal hypospadias continues, two-stage or single-stage, and neither method is accepted universally. In terms of the current technical level, the vast majority of severe hypospadias operations can be performed in one stage [4, 5], and Duckett [6] and Koyanagi [7] urethroplasties are currently the most commonly used one-stage procedures. For patients with severe hypospadias (treated initially) and failed hypospadias, a staged operation may be a wise choice. There are many two-stage operations, such as Byar’s operation [8], Bracka’s operation [9, 10], staging Duckett surgery [11], and free graft roll tube surgery [12, 13]. At present, in the first stage of staged surgery, the penis should be fully extended, and part of the urethra should be reconstructed; this approach is different from previous staging surgeries. The choice of surgical treatment for severe hypospadias should be different from person to person, emphasizing individual treatment and objectively choosing a one-stage or two-stage operation to achieve the best therapeutic effect.

We have performed one-stage and two-stage urethroplasty combining transected urethral plate and TPIF to treat initial severe hypospadias in our hospital since 2015. We observed a success rate of 61.3% for one-stage TPIF urethroplasty and 95.8% for staged TPIF urethroplasty from August 2015 to December 2019, and there was statistical significance in the incidence of success rate between the two groups. The incidence of urinary fistula and urethral diverticulum after staging operation was significantly decreased compared with that of one-stage operation, and the differences between the two groups were statistically significant.

Almost all severe hypospadias cases have different degrees of chordee [14, 15], especially the Donnahoo IV type form [16], which has been described as a developmental defect in the distal urethra of fibrous shortening. The difficulty of correction involves not only cutting off the developmental defects of the fibrous urethra to fully correct the bending deformity but also selecting the appropriate self-tissue to reconstruct the urethra. Therefore, when we treat severe hypospadias, the urethral plate should be transected after the fibrous cord on the ventral surface of the cavernous body of the penis is released [17], and dorsal plications should be used to straighten the penis and completely correct the ventral penile curvature. Schlomer [18] proposed that mild-to-moderate residual ventral curvature of the penis after transection of the urethral plate should be corrected with dorsal plication, while severe ventral penile curvature should be corrected with ventral lengthening. In the two groups of patients, dorsal plication was used to correct residual chordee after transection of the urethral plate, with satisfactory results, and no ventral lengthening of the penis was used. No cases of residual or recurrence of penile chordee were reported in the two groups.

Urethrocutaneous fistula easily occurs after hypospadias repair. The occurrence of a urethrocutaneous fistula is closely related to the blood supply of the urethral flap and the quality of the waterproof layer between the urethra and the skin. Since the length of the urethroplasty at the first stage of Group B has no strict requirement, the surgeon can select the part with the best vascular conditions of the island flap to form the urethra, which is conducive to the healing of the urethra and thus reduces the incidence of urinary fistulas. In addition, the urethral tube should be formed at the first stage with one end opening at the scaphoid fossa to create an external urethral orifice and the other end opening between the scrotum. Both ends of the urethral tube have openings, forming a good drainage channel. Even if the wound produces postoperative secretions, they can flow out from both ends of the formed urethra relatively easily, avoiding the accumulation of secretions in the wound and affecting wound healing, which is conducive to reducing the occurrence of a urinary fistula. In the present study, during the second operation, the operative site was located in the scrotum, and the formed urethra was covered with rich dartos tissue or a tunica vaginalis flap, which greatly reduced the incidence of urethral fistula. In agreement with the findings of Pescheloche et al., using the tunica vaginalis flap as an intermediate layer to cover the neourethra can effectively reduce postoperative complications [19].

Inconsistent with the findings of Tiryaki et al., modification of the Bracka technique using a flap for the urethral plate resulted in a high incidence of diverticulum formation [20]. No urethral diverticula occurred in Group B (how to reduce or avoid complications is the goal we pursue). One reason is that the pedicled island flap was taken from the relatively flat prepuce portion, and the width of the flap was appropriate for preventing the new urine tube from being too loose or too tight; another reason is that staged TPIF urethroplasty enables voiding through the stoma after the first stage, which can effectively reduce the impact of high pressure flow on the distal newly formed urethra in the early postoperative period. This procedure provided at least 6 months for the reconstructed neourethra to gradually adhere to the corpora and surrounding fascial tissue, and the outer wall of the urethra was well supported, avoiding the above risk factors for the formation of urethral diverticula.

Urethral stricture is a serious complication after proximal hypospadias repair and commonly occurs at the urethra anastomosis site in one-stage TPIF urethroplasty. In recent years, some scholars [21] have advocated that a three-week instead of one-week catheterization and an age below 6 months at hypospadias repair are associated with a better outcome and fewer complications. Urethral stricture often occurs 2–4 weeks after removal of the catheter. Prolonging the dwelling time of the urinary catheter is beneficial for the subsidence of inflammatory reactions and edema in the operative area, reduction in the occurrence of urinary extravasation, and passage through the urethral scar period. At the same time, the catheter provides better support for the newly formed urethra, which can avoid urethral angulation and distortion and reduce the occurrence of urethral strictures. In addition, expansion of the glans during the first stage of the operation can also effectively prevent the risk of postoperative metal stenosis or glans dehiscence. In the two groups, the urethral catheter was removed 3 weeks after the operation, and none of the patients developed narrowing of the new urethra in Group B.

The limitations of our study were the absence of urodynamic tests and the absence of an objective scoring system to evaluate postoperative urination.

## Conclusions

In summary, staged surgery should be considered for the correction of severe hypospadias, especially when repair materials are insufficient, penile dysplasia combined with many deformities is present, and surgical experience is inadequate. Staged TPIF urethroplasty has a low incidence of total complications compared with one-stage TPIF urethroplasty. The staged TPIF urethroplasty procedure was effective in reducing the operation difficulty and complication rate of severe hypospadias, improving the curative effect of complex hypospadias and having good clinical application value.

## Data Availability

The datasets used and analysed during the current study are available from the corresponding author on reasonable request.

## Abbreviation

TPIF: Transverse preputial island flap
